# Average Power Handling Capability of Corrugated Slow-Wave Transmission Lines

**DOI:** 10.3390/mi13060961

**Published:** 2022-06-17

**Authors:** Zehao Zheng, Min Tang, Haochi Zhang, Junfa Mao

**Affiliations:** 1 Key Laboratory of Ministry of Education of China for Research of Design and Electromagnetic Compatibility of High Speed Electronic Systems, Shanghai Jiao Tong University, Shanghai 200240, China; zzh1996@sjtu.edu.cn (Z.Z.); jfmao@sjtu.edu.cn (J.M.); 2 State Key Laboratory of Millimeter Waves, Southeast University, Nanjing 210096, China; hczhang@etnws.com

**Keywords:** average power handling capability (APHC), slow-wave transmission line (SWTL), thermal resistance, average heat-spreading width, temperature-dependent resistivity

## Abstract

In this article, the average power handling capability (APHC) of corrugated slow-wave transmission lines (SWTLs) is investigated. Firstly, the attenuation constants of conductor and dielectric are extracted by the multiline method. Secondly, the thermal resistance of corrugated SWTLs is analyzed based on the constant-angle model. To deal with the non-uniform corrugated structure of SWTLs, the concept of average heat-spreading width (AHSW) is introduced. Finally, the APHC of the corrugated SWTL is calculated using the attenuation constant and the thermal resistance. In addition, the APHC considering the temperature-dependent resistivity of metal conductor is also presented. For validation, the APHCs of SWTLs with different geometric parameters are evaluated. The results agree well with those obtained by the commercial software.

## 1. Introduction

Corrugated slow-wave transmission lines (SWTLs), as a kind of TLs with periodic structures, have attracted much attention in the microwave area. With the advantage of controllable dispersion properties, the corrugated SWTLs can achieve a specific phase delay without changing the longitudinal length, which brings much convenience to the circuit design. In recent years, corrugated SWTLs are popular candidates in the design of microwave circuits, such as miniaturized antennas [1,2,3], miniaturized power dividers [4], antenna feeding networks [5], and broadband baluns [6].

With the increase of integration level and the trend of miniaturization of microwave components, thermal effect is becoming an essential issue that must be considered during the design of microwave systems. In order to avoid thermal failure, the operating temperature of the microwave system should be controlled. Thus, the power transmitted into the system should be limited below the average power handling capability (APHC). The APHC of a microstrip line is first studied, where the parallel-plate model is proposed to calculate the temperature rise [7]. The parallel-plate model is also utilized in estimating the APHCs of multilayer microstrip lines [8] and various microwave passive components [9,10,11]. However, for the transmission lines with more complicated structures, such as coupled microstrip lines [12,13] and coplanar waveguides [14,15], the constant-angle model is more often used when estimating the APHCs. Although the SWTLs are utilized in the design of various microwave circuits, to the best of the authors’ knowledge, their APHCs have not yet been investigated.

In this article, the APHCs of corrugated SWTLs are estimated using the constant-angle model. The thermal resistances of the conductor and dielectric are first analyzed. The concept of average heat-spreading width (AHSW) is employed in the derivation of thermal resistance of the corrugated structure. The effect of temperature-dependent resistivity of metal conductor is also considered. Using the developed closed-form expressions, the APHCs of corrugated SWTLs with different geometric parameters are evaluated efficiently. The validity of the proposed model is verified by the commercial software.

## 2. Analysis of the Corrugated Slow-Wave Transmission Lines

### 2.1. Dispersion Characteristics

**Figure 1:**
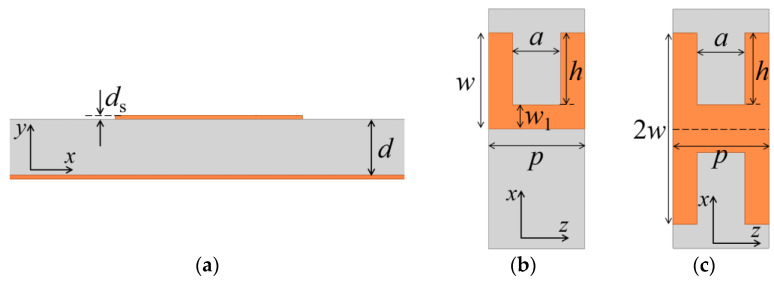
Configuration of the corrugated SWTL: (**a**) cross-sectional view; (**b**) top view of the U-shaped unit; (**c**) top view of the H-shaped unit.

The configuration of typical corrugated SWTLs is described in Figure 1. The unit structure may be U-shaped or H-shaped. The top view of the U-shaped unit is shown in Figure 1b, with the groove width *a*, the groove length *h*, the period length *p*, the total width *w*, and the width of main line *w*_1_ (*w*_1_ = *w* − *h*). The H-shaped unit is obtained by a mirror transformation of the U-shaped unit, as is shown in Figure 1c, where the dashed line is the axis of symmetry.

**Figure 2:**
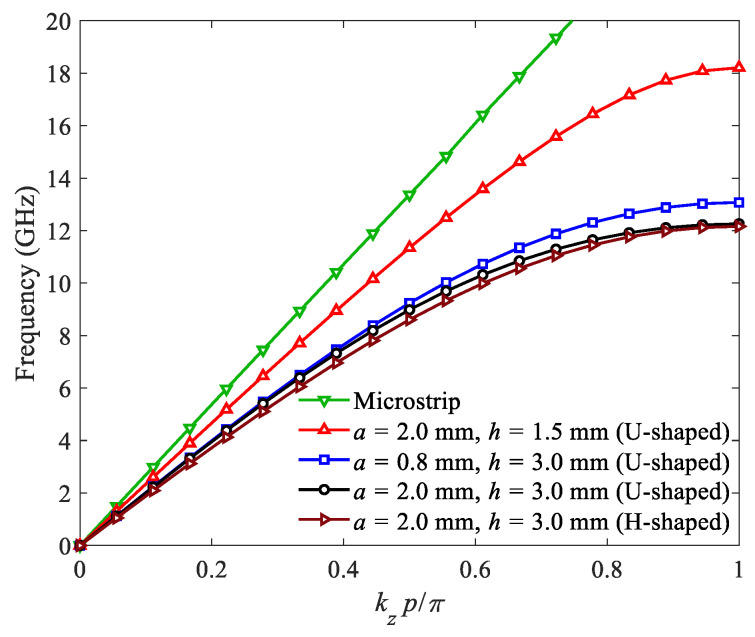
Dispersion curves of the corrugated SWTLs.

The dispersion curves of the corrugated SWTLs are obtained by the eigenmode simulation, as shown in Figure 2, where *k_z_* is the wavenumber along the *z* axis. The dimensions of the corrugated SWTLs are *p* = 4 mm, *w*_1_ = 1 mm, *d* = 0.508 mm, *d_s_* = 0.018 mm and the relative permittivity of the substrate is *ε_r_* = 2.2. For comparison, the dispersion curve of a 50 Ω microstrip line is also provided. It is observed that the wavenumbers of the corrugated SWTLs are larger than that of the microstrip line within the whole frequency range, exhibiting an obvious slow-wave characteristic. For the U-shaped units, the dispersion curve deviates from the microstrip line as the groove length *h* increases. The increase of asymptotic frequency is observed as the groove width *a* decreases. In addition, it is found that the dispersion curves of the H-shaped unit and U-shaped unit with the same geometric parameters almost coincide, where the wavenumber of the H-shaped unit is slightly larger.

### 2.2. Extraction of Conductor and Dielectric Attenuation

**Figure 3:**
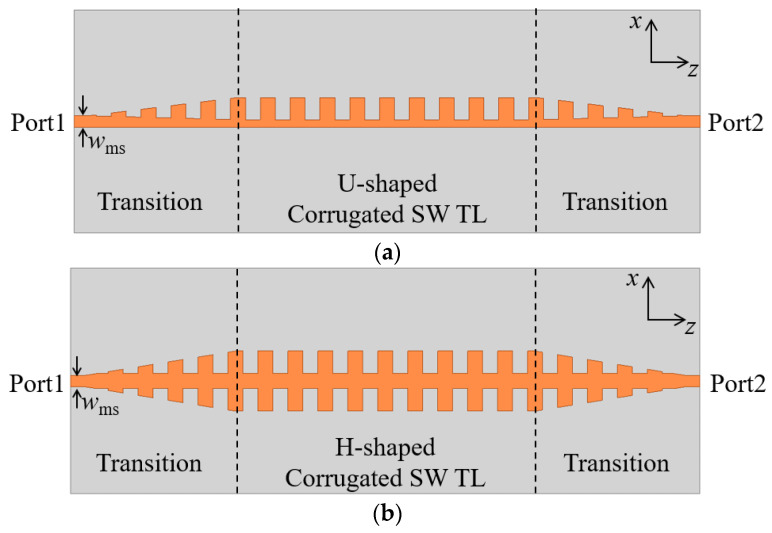
Configuration of the corrugated SWTLs with transitions: (**a**) U-shaped; (**b**) H-shaped.

The dispersion curves in Figure 2 show that the wavenumbers of the corrugated SWTLs are larger than that of the microstrip, and the deviation is relieved with the decrease of *h*. Therefore, to compensate for the impedance and momentum mismatch between the microstrip and SWTL, the transition regions are designed with gradient corrugations, as described in Figure 3.

To accurately characterize the APHC of the corrugated SWTLs, the conductor and dielectric attenuation constants should be extracted first. For the purpose of eliminating the impact of transition regions, the multiline method [16] is utilized. In this method, two corrugated SW structures with different numbers of units are investigated. The total transmission matrices of them can be written as

(1) $$T_{1}=T_{\mathrm{left}}T_{N1}T_{\mathrm{right}}$$

(2) $$T_{2}=T_{\mathrm{left}}T_{N2}T_{\mathrm{right}}$$

where **T**_*N*1_ and **T**_*N*2_ are the transmission matrices of the SWTLs with the unit number of *N*_1_ and *N*_2_, respectively. The transmission matrices of the transition regions on the left and right sides are represented by **T**_left_ and **T**_right_, respectively.

From (1) and (2), we obtain

(3) $$T_{12}T_{\mathrm{left}}=T_{\mathrm{left}}T_{\mathrm{DUT}}$$

where

(4) $$T_{12}=T_{1}T_{2}^{-1}$$

(5) $$T_{\mathrm{DUT}}=T_{N1}T_{N2}^{-1}=\left[\begin{array}{cc} e^{\gamma \left(N_{2}-N_{1}\right)p} & 0\\ 0 & e^{-\gamma \left(N_{2}-N_{1}\right)p} \end{array}\right]$$

where *γ* is the complex propagation constant.

Thus, the total attenuation constant is derived by

(6) α=Re(γ)=Re{1|N2−N1|pln[12(λ1+1λ2)]}

where *λ*_1_ and *λ*_2_ (|*λ*_1_|>|*λ*_2_|) are the two eigenvalues of **T**_12_ matrix.

Note that with the special settings of material, the conductor attenuation constant *α_c_* and the dielectric attenuation constant *α_d_* can be extracted separately based on the above technique. For example, when the loss of metal is omitted, the dielectric attenuation constant *α_d_* can be extracted alone. Similarly, the conductor attenuation constant *α_c_* can be obtained if the loss of substrate is ignored.

## 3. Modeling of Thermal Resistance

### 3.1. Thermal Resistance for U-Shaped Corrugated SWTL

**Figure 4:**
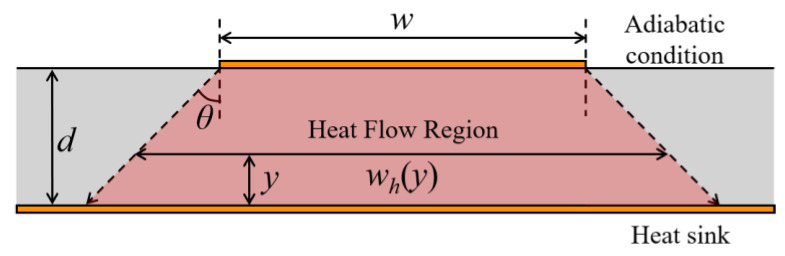
Cross-sectional view of the heat flow region in the constant-angle model.

Assume that the ground plane of SWTL is the heat sink with constant temperature and the other boundaries are adiabatic, as shown in Figure 4. According to the constant-angle model [17,18], the heat flow region locates within a heat-spreading angle *θ*, and *w_h_*(*y*) is the width of the heat flow region at height *y*.

Thus, the thermal resistances for a transmission line per unit length can be derived as

(7) $$R_{thc}=\frac{1}{K}\int_{0}^{d}\frac{dy}{w_{h}\left(y\right)}$$

(8) $$R_{thd}=\frac{1}{K}\int_{0}^{d}\frac{\left(1-y/t\right)dy}{w_{h}\left(y\right)}$$

where *R_thc_* and *R_thd_* are the thermal resistances of the conductor and dielectric, respectively, and *K* is the thermal conductivity of the substrate. 

**Figure 5:**
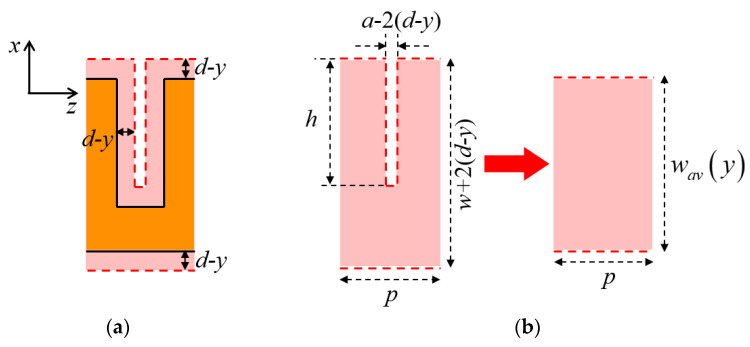
Top view of the heat flow region of U-shaped corrugated SWTL: (**a**) Boundary of the heat flow region; (**b**) schematic of *w_av_*(*y*).

For the U-shaped corrugated SWTLs, the top view of the heat flow region is shown in Figure 5. Applying *θ* = 45°, the horizontal distance between the boundary of the heat flow region and the metal conductor is (*d* – *y*) at the height of *y*, as shown in Figure 5a. As a result, *w_h_*(*y*) varies along the propagation direction (*z*-axis) because of the non-uniform corrugated structure of the conductor, making it difficult to calculate the thermal resistance.

To overcome this problem, the concept of AHSW is introduced in this work, where the heat flow region of a unit U-shaped SWTL is replaced by a uniform rectangular region with the same area and longitudinal length, as shown in Figure 5b. The width *w_av_*(*y*) of the rectangular region is defined as AHSW.

In the situation of *d* ≤ *a/*2, a rectangular gap always exists in the heat flow region. Therefore, the AHSW can be expressed as

(9) $$w_{av}\left(y\right)=w+2\left(d-y\right)-\left[a-2\left(d-y\right)\right]h/p$$

Otherwise, the rectangular gap in the heat flow region vanishes when *y* < (*d* − *a*/2), and the expression of AHSW is

(10) $$w_{av}\left(y\right)=\{\begin{array}{cc} w+2\left(d-y\right), & 0<y<d-a/2\\ w+2\left(d-y\right)-\left[a-2\left(d-y\right)\right]h/p, & d-a/2\leq y<d \end{array}$$

Then, replacing *w_h_*(*y*) by *w_av_*(*y*) in (7) and (8), the thermal resistances for the U-shaped corrugated SWTLs are

(11) $$R_{thc}=\{\begin{array}{cc} \frac{1}{2K\left(1+h/p\right)}\ln\left(1+\frac{2d\left(1+h/p\right)}{w-ah/p}\right), & d\leq a/2\\ \frac{1}{2K}\left(\ln\frac{w+2d}{w+a}+\frac{1}{1+h/p}\ln\frac{w+a}{w-ah/p}\right), & d>a/2 \end{array}$$

(12) $$R_{thd}=\{\begin{array}{cc} \frac{1}{2K\left(1+h/p\right)}\left[1-\frac{w-ah/p}{2d\left(1+h/p\right)}\ln\left(1+\frac{2d\left(1+h/p\right)}{w-ah/p}\right)\right], & d\leq a/2\\ \begin{array}{l} \frac{1}{2K}[\left(1-\frac{a}{2d}-\frac{w}{2d}\ln\frac{w+2d}{w+a}\right)\\ +\frac{1}{1+h/p}\left(\frac{a}{2d}-\frac{w-ah/p}{2d\left(1+h/p\right)}\ln\frac{w+a}{w-ah/p}\right)], \end{array} & d>a/2 \end{array}$$

### 3.2. Thermal Resistance for H-Shaped Corrugated SWTL

**Figure 6:**
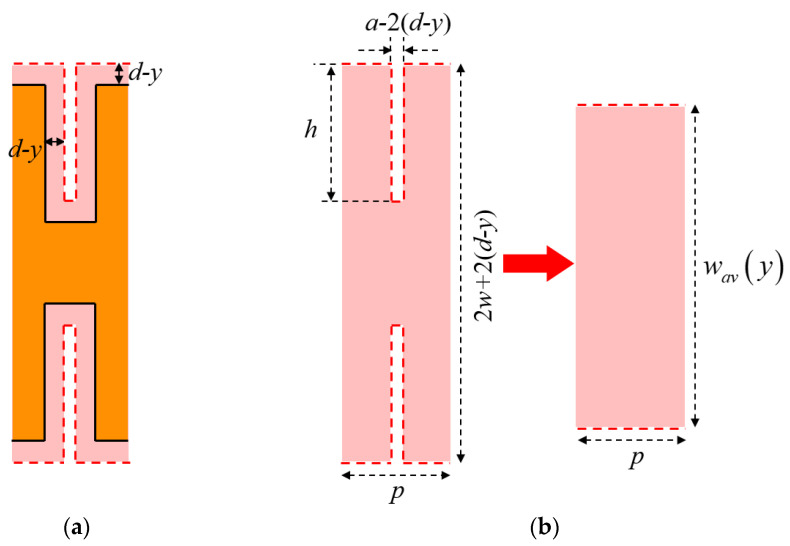
Top view of the heat flow region of H-shaped corrugated SWTL: (**a**) Boundary of the heat flow region; (**b**) schematic of *w_av_*(*y*).

Applying the same constant-angle model with *θ* = 45°, the heat flow region of a H-shaped corrugated SWTL is shown in Figure 6. The derivation of AHSW is similar to that of the U-shaped one, as is shown in Figure 6b.

When *d* ≤ *a/*2, the AHSW of the H-shaped corrugated SWTL can be expressed as

(13) $$w_{av}\left(y\right)=2w+2\left(d-y\right)-2\left[a-2\left(d-y\right)\right]h/p$$

Otherwise,

(14) $$w_{av}\left(y\right)=\{\begin{array}{cc} 2w+2\left(d-y\right), & 0<y<(d-a/2)\\ 2w+2\left(d-y\right)-2\left[a-2\left(d-y\right)\right]h/p, & (d-a/2)\leq y<d \end{array}$$

Comparing (13)–(14) with (9)–(10), it is observed that the AHSW of the H-shaped corrugated SWTL can be obtained from the U-shaped one by substituting *w* and *h* with 2*w* and 2*h*. Moreover, the thermal resistances for the H-shaped corrugated SWTLs can be obtained with the same substitution.

## 4. Derivation of Average Power Handling Capability

For a lossy transmission line, the propagated power along the line satisfies the following expression

(15) $$P\left(z\right)=P_{0}e^{-2\alpha z}$$

where *P*_0_ is the input power. Using the first-order approximation, the power loss per unit length is calculated as

(16) $$\Delta P=\frac{P(0)-P(l)}{l}=\frac{P_{0}\left(1-e^{-2\alpha l}\right)}{l}\approx 2\alpha P_{0}\alpha$$

where *l* is the length of the transmission line. As the power loss is treated as heat source, the temperature rise per unit input power of the SWTL is calculated as

(17) $$\Delta T=\frac{\Delta P_{c}R_{thc}+\Delta P_{d}R_{thd}}{P_{0}}=2\alpha_{c}R_{thc}+2\alpha_{d}R_{thd}$$

where Δ*P_c_* and Δ*P_d_* are the power loss due to conductor and dielectric attenuation.

Thus, the APHC of the corrugated SWTL can be calculated by

(18) $$P_{av}=\frac{T_{max}-T_{amb}}{\Delta T}=\frac{T_{max}-T_{amb}}{2\alpha_{c}R_{thc}+2\alpha_{d}R_{thd}}$$

where *T_max_* is the maximum operating temperature, and *T_amb_* is the ambient temperature.

In practice, the resistivity of metal conductor usually changes with temperature, resulting in the variation of conductor attenuation. In order to take this impact into account, the expression of APHC in (18) need to be modified. The temperature-dependent resistivity of most metals is expressed by the following linear approximation [19]

(19) $$\rho \left(T\right)=\rho_{0}\left[1+\alpha_{T}\left(T-T_{amb}\right)\right]$$

where *ρ*_0_ is the resistivity at *T_amb_*, and *α_T_* is the temperature coefficient of resistivity. 

As the conductor attenuation is proportional to the square root of resistivity [20], the impact of temperature on the conductor attenuation is expressed by

(20) $$\alpha_{c}\left(T\right)=\alpha_{c}{}_{0}\sqrt{1+\alpha_{T}\left(T-T_{amb}\right)}$$

where *α_c_*_0_ is the conductor attenuation at *T_amb_*.

Thus, considering the effect of temperature-dependent resistivity, the APHC is modified as

(21) $$P_{av}=\frac{T_{max}-T_{amb}}{2\alpha_{c}{}_{0}\sqrt{1+\alpha_{T}\left(T_{max}-T_{amb}\right)}R_{thc}+2\alpha_{d}R_{thd}}$$

## 5. Numerical Results and Discussion

### 5.1. Temperature Rise of U-Shaped Corrugated SWTL

In this section, the temperature rise of the U-shaped corrugated SWTL is evaluated using the proposed model. The substrate is Rogers RT5880 (*ε_r_* = 2.2, tan*δ* = 0.0009, *K* = 0.2 W/(m°C)) with the thickness of 0.508 mm. The metal conductor is copper (*σ* = 5.8 × 10^7^ S/m) with the thickness of 0.018 mm. The effect of temperature-dependent resistivity is not considered initially. The geometrical parameters of the U-shaped corrugated SWTLs are listed in Table 1, where three samples with different values of groove width *a* and groove length *h* are investigated.

Firstly, the thermal resistances of the three samples are calculated by (11) and (12), and the results are given in Table 2. It is observed that both *R_thc_* and *R_thd_* increase with the decrease of *h* and the increase of *a*.

**Figure 7:**
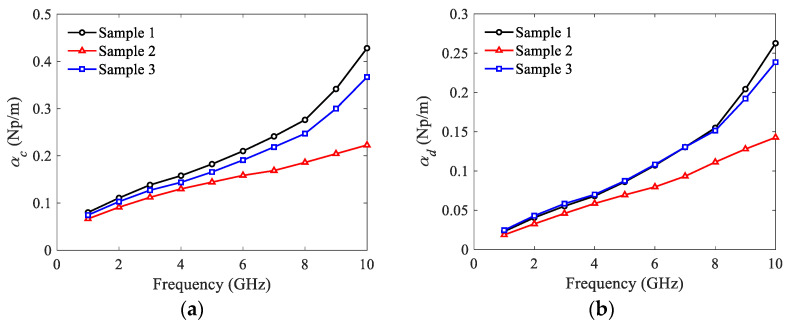
Attenuation constants of U-shaped corrugated SWTLs: (**a**) conductor attenuation; (**b**) dielectric attenuation.

Then, the attenuation constants of the samples are extracted. The results of conductor and dielectric attenuation constants as functions of frequency are depicted in Figure 7. It is observed that the groove width *a* has small impact on the attenuation constants, especially for the dielectric attenuation case. In contrast, with the increase of operating frequency, the impact of groove length *h* is significant on the attenuation constants of SWTLs.

**Figure 8:**
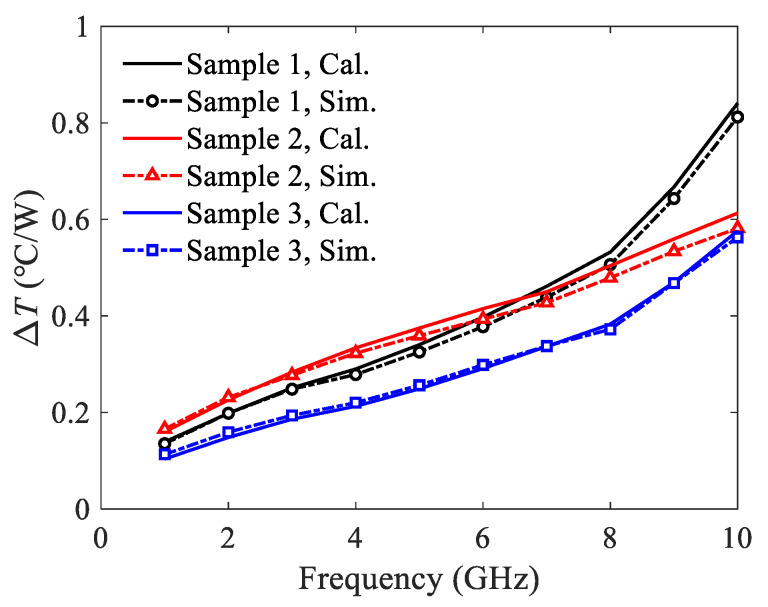
Temperature rises per watt of the U-shaped corrugated SWTLs.

Based on the above results of thermal resistances and attenuation constants, the temperature rises can be calculated directly by (17). To validate the proposed model, the temperature rises are also simulated using a commercial solver ANSYS. The input power is 50 W, and the ground plane is treated as heat sink with a fixed temperature of *T_amb_* = 25 °C. The temperature rises per watt are shown in Figure 8. Good agreement is observed between the calculated results and the simulated ones.

In Figure 8, there is an intersection in the simulated results of sample 1 and sample 2 at around 6.5 GHz. A brief explanation of this phenomenon is given as below. The thermal resistance of sample 1 is smaller, which leads to the less temperature rise per watt in the low frequency range. However, the attenuation constant of sample 1 is much larger than that of sample 2 at high frequencies. Therefore, the resulting temperature rise per watt is higher. In addition, the temperature rise per watt of sample 3 is the smallest due to its lowest thermal resistance.

**Figure 9:**
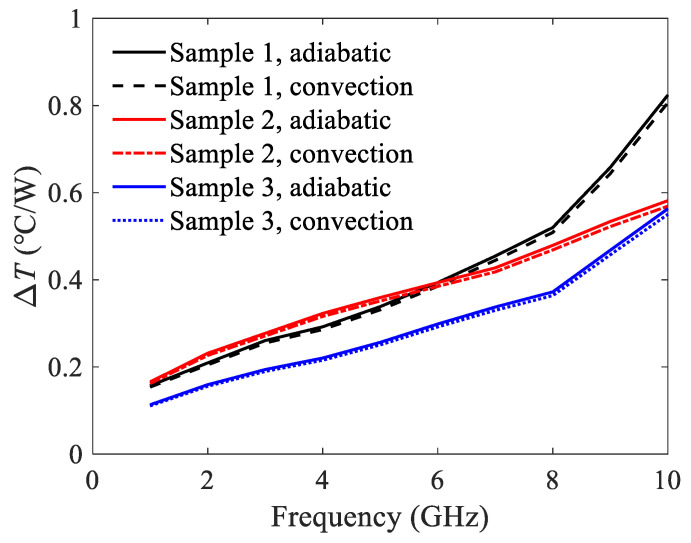
Temperature rises per watt of the U-shaped corrugated SWTLs when the top surface is under adiabatic or natural convection condition.

It should be mentioned that for all the above results, the top surfaces of the structures are assumed to be adiabatic. In practice, however, there is convective heat transfer from the top surface to the ambient. In order to investigate the effect of heat convection on the top surface, the temperature rise of the samples are simulated by ANSYS, where the convection coefficient is set to 10 W/(m^2^°C) to represent the natural convection situation. The results are compared with those under the adiabatic assumption, as shown in Figure 9. It is observed that the temperature rise per watt will not be affected apparently when the natural convection condition is imposed. Therefore, the heat convection on the top surface is negligible and the assumption of adiabatic condition is reasonable.

**Figure 10:**
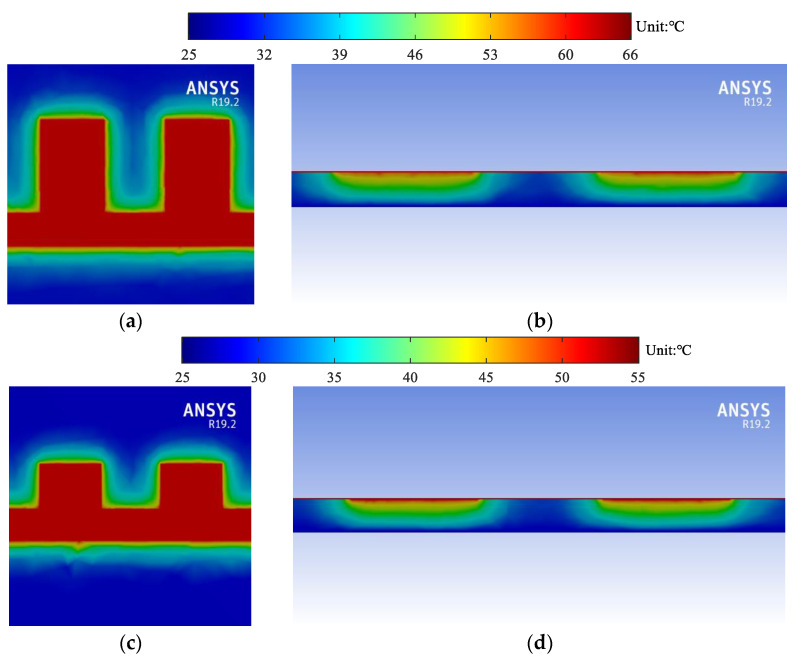

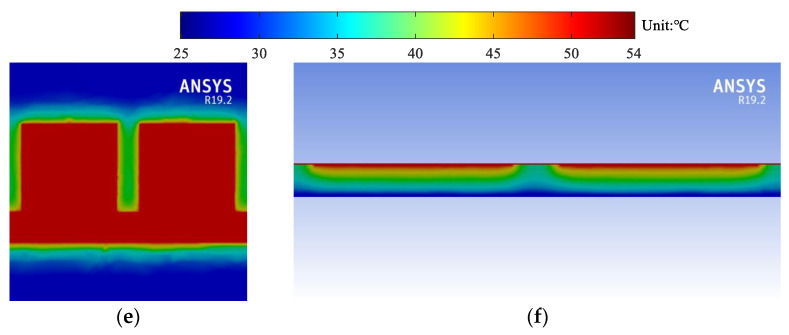
Simulated thermal profile of the U-shaped corrugated SWTLs: (**a**) top view of sample 1; (**b**) cross-sectional view of sample 1; (**c**) top view of sample 2; (**d**) cross-sectional view of sample 2; (**e**) top view of sample 3; (**f**) cross-sectional view of sample 3.

For more detailed illustration, the thermal profiles at 10 GHz are shown in Figure 10. It is shown that the maximum temperature of sample 1 is the highest. The maximum temperature of sample 2 is lower due to the decrease of attenuation constants, while that of sample 3 is the lowest due to the smallest thermal resistance.

### 5.2. Temperature Rise of H-Shaped Corrugated SWTL

The temperature rise of the H-shaped corrugated SWTL is studied in this section. The material of conductor and dielectric are the same as the U-shaped ones, and the geometric parameters are listed in Table 3. Note that this H-shaped corrugated SWTL can be formed by the mirror transformation of the U-shaped one (sample 1) in the previous section.

The thermal resistances of the H-shaped corrugated SWTL are calculated using (11) and (12) by replacing *w* and *h* with 2*w* and 2*h*, as shown in Table 4. For comparison, the thermal resistances of the U-shaped SWTL (sample 1) are also listed in Table 4. The results show that the thermal resistances of the H-shaped SWTL are lower than those of the U-shaped one.

**Figure 11:**
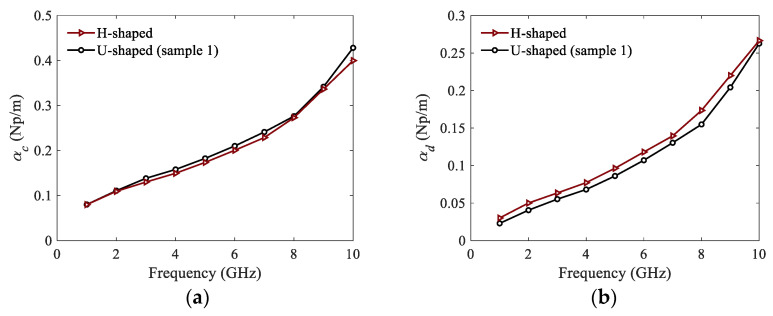
Attenuation constants of the corrugated SWTLs: (**a**) conductor attenuation; (**b**) dielectric attenuation.

The conductor and dielectric attenuation constants of the H-shaped and U-shaped corrugated SWTLs are simulated and plotted in Figure 11. It is observed that the conductor attenuation constant of the H-shaped SWTL is very close to that of the U-shaped one. Moreover, the dielectric attenuation constant of the H-shaped SWTL is slightly higher than that of the U-shaped one because of the larger wavenumber depicted in Figure 2.

**Figure 12:**
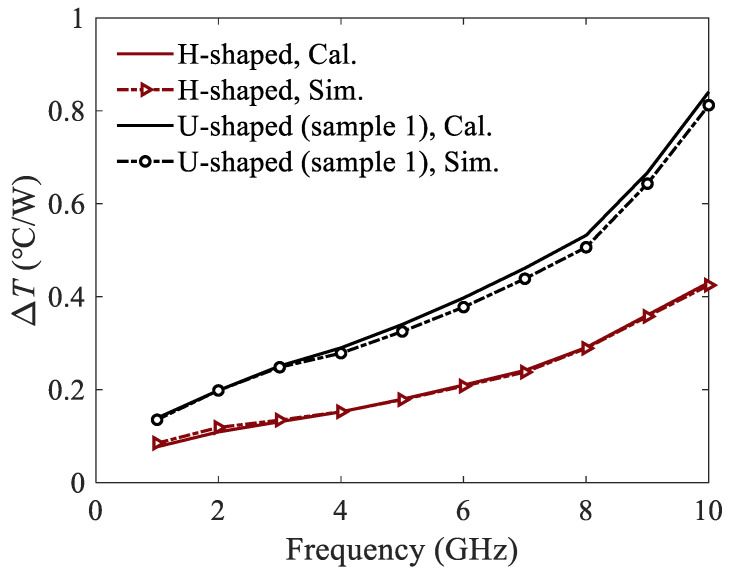
Temperature rises per watt of the corrugated SWTLs.

**Figure 13:**
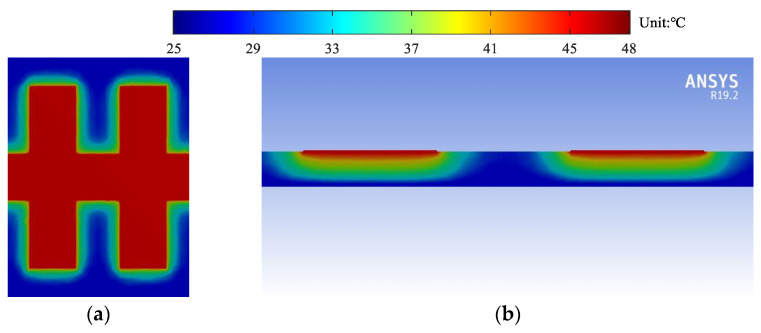
Simulated thermal profile of the H-shaped corrugated SWTL: (**a**) top view; (**b**) cross-sectional view.

Similarly, the temperature rises of the H-shaped and U-shaped corrugated SWTLs are calculated by (17) and simulated by ANSYS, as shown in Figure 12. The thermal profile of the H-shaped corrugated SWTL is shown in Figure 13, where the simulation condition is the same as those for the U-shaped ones. It is observed that the temperature rise of the H-shaped SWTL is obviously lower than the U-shaped one, which is due to the similar attenuation constants but smaller thermal resistances. Furthermore, the calculated results agree well with the simulated ones, proving the validity of the proposed model.

### 5.3. APHCs of the Corrugated SWTLs

When the temperature rise is known, the APHCs of the corrugated SWTLs are readily obtained by (18). To consider the impact of temperature-dependent resistivity, the APHCs should be calculated using (21), where the temperature coefficient of resistivity for copper is *α_T_* = 0.0039 °C^−1^. For validation, the APHCs are also obtained by the electro-thermal co-simulation of ANSYS. The results at the operating frequency of 10 GHz are compared in Table 5. It is observed that the APHC of the H-shaped corrugated SWTL is the highest, due to the smallest thermal resistances. Among the U-shaped ones, sample 1 has the lowest APHC because of the largest attenuation constants. Sample 2 has a higher APHC due to the smaller attenuation constant, and sample 3 holds the highest APHC because of the smallest thermal resistance.

Furthermore, the calculated APHCs with the consideration of temperature-dependent resistivity are in accordance with the results of ANSYS, where the maximum relative error is less than 5%. In contrast, the APHCs will be overestimated when the temperature-dependent resistivity is ignored, and the maximum relative error is up to 16.0%. Therefore, the consideration of temperature-dependent resistivity is rather necessary

## 6. Conclusions

The closed-form expressions to estimate the APHCs of corrugated SWTLs are provided in this article. The thermal resistances of both U-shaped and H-shaped SWTLs are calculated based on the constant-angle model, where the AHSW is introduced to deal with the non-uniform corrugated structure. The temperature-dependent resistivity of the metal conductor is also considered in the evaluation of APHC. Good agreement of the APHC results is achieved from the proposed model and the commercial software.

## Figures and Tables

**Table 1:** Geometric parameters of the U-shaped corrugated SWTLs (unit: mm).

	*a*	*h*	*p*	*w*_1_
Sample 1	2	3	4	1
Sample 2	2	1.5	4	1
Sample 3	0.8	3	4	1

**Table 2:** Thermal resistances of U-shaped corrugated SWTLs (unit: m°C/W).

	Sample 1	Sample 2	Sample 3
*R_thc_*	0.7674	1.0670	0.6027
*R_thd_*	0.3495	0.4816	0.2811

**Table 3:** Geometric parameters of the H-shaped corrugated SWTL (unit: mm).

*a*	*h*	*p*	*w*
2	3	4	4

**Table 4:** Thermal resistances of corrugated SWTL (unit: m°C/W).

	H-Shaped	U-Shaped (Sample 1)
*R_thc_*	0.4108	0.7674
*R_thd_*	0.1914	0.3495

**Table 5:** APHCs of the corrugated SWTLs.

	ANSYS	Proposed Model		Proposed Model (TDR ^1^)	
	APHC (W)	APHC (W)	RE ^2^	APHC (W)	RE
U-shaped (sample 1)	78.5	89.2	13.6%	80.6	2.6%
U-shaped (sample 2)	105.5	122.4	16.0%	110.6	4.9%
U-shaped (sample 3)	115.3	130.1	12.8%	117.8	2.1%
H-shaped	153.9	174.2	13.2%	157.8	2.5%

^1^ TDR: temperature-dependent resistivity.^2^ RE: relative error.
